# Ectopic prolactin secretion secondary to an ovarian tumour

**DOI:** 10.1530/EDM-13-0016

**Published:** 2013-08-30

**Authors:** Sowmya Gururaj, K Nisal, Q Davies, S Deen, P G McNally

**Affiliations:** University Hospitals of LeicesterLeicesterUK; 1 Nottingham University HospitalNottinghamUK

## Abstract

Ectopic hormone secretion is a well-recognised phenomenon; however, ectopic prolactin secretion is exceptionally rare. Hoffman and colleagues reported the first ever well-documented case of ectopic prolactin secretion secondary to a gonadoblastoma. We report a lady who presented with galactorrhoea and a large ovarian tumour that was found to secrete high levels of prolactin.

**Learning points:**

- Aim of this case report is to highlight the occurrence of this condition.
- Lack of awareness can often lead to a diagnostic conundrum.

## Background

We report this case to make clinicians aware of this rare condition (1) (2). Hoffman and colleagues reported the first ever well-documented case of ectopic prolactin secretion secondary to a gonadoblastoma (1).

## Case presentation

A 27-year-old lady presented with 8-week history of hot flushes, headaches, galactorrhoea and delayed periods. She denied any problems with vision or fluctuation in weight. Her medical history was asthma and irritable bowel disease. She was an ex-smoker of 5 years with a two packs per year smoking history and drank alcohol occasionally. On examination, she was noted to have galactorrhoea on expression. She had normal visual fields tested by confrontation method. Systemic examination revealed a 10 cm supra-pubic mass on palpation.

## Investigations

### Biochemistry

Her initial prolactin level returned at 24 642 mIU/l (normal range 50–400 mIU/l) with suppressed LH <0.5 IU/l, FSH 2.7 IU/l and 17β-oestradiol (E_2_) 83 pmol/l. She had normal TSH 1.8 mIU/l (0.30–5.00), thyroxine 12 pmol/l (9–25 pmol/l) and normal cortisol response to short synacthen test (basal 251- and 30-min levels of 753 nmol/l). Her tumour markers showed CA 125 antigen 67 kU/l (0–35), alphafetoprotein 83 kU/l (0–10), CA19-9 antigen 19 kU/l (0–10) and carcino-embryonic antigen <2 (0–5). Her repeat prolactin level was 36 066 mIU/l.

### Imaging

**Figure 1:**
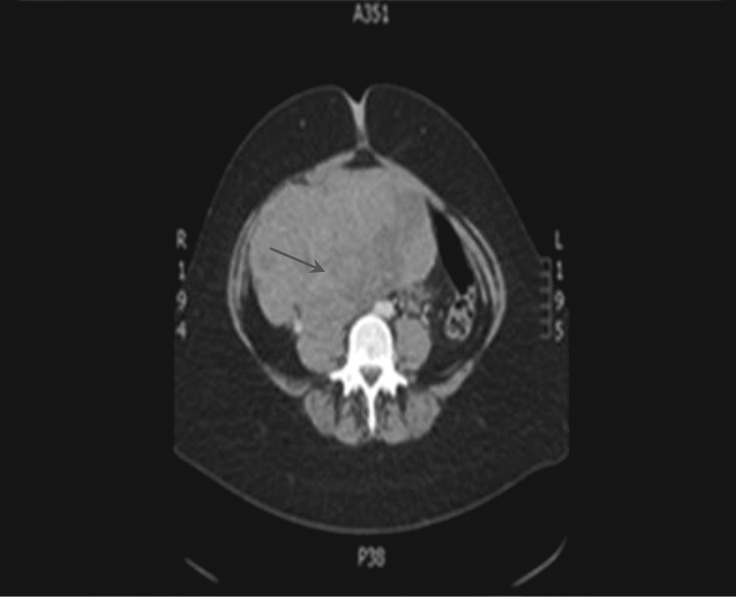
CT abdomen showing large right adnexal mass (red arrow).

Magnetic resonance imaging of the pituitary was normal. Her computed tomography (CT) of the abdomen showed a right adnexal mass measuring 18×14×18 cm with retroperitoneal lymphadenopathy (Fig. 1).

## Treatment

**Figure 2:**
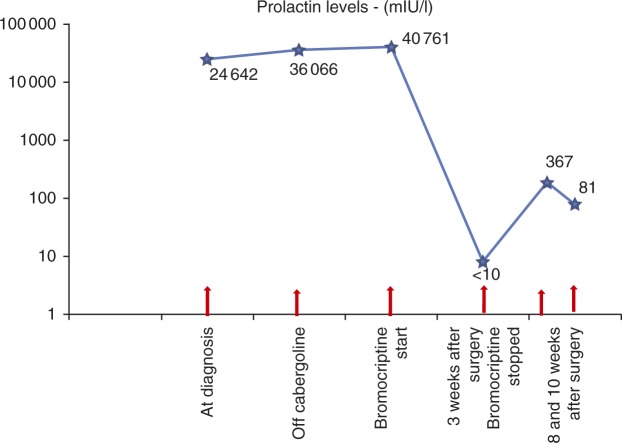
Prolactin levels (mIU/l).

Therapy with cabergoline was initiated but discontinued due to intolerance. She was then started on bromocriptine 2.5 mg. She underwent right salpingo-oophorectomy with para-aortic node clearance. Three weeks post-operatively, her prolactin dropped to undetectable levels. Bromocriptine was discontinued. Her prolactin levels remained normal (Fig. 2) at 367 and 81 mIU/l with a normal LH 2.2, FSH 2.8 and E_2_ 355 pmol/l.

## Outcome and follow-up

**Figure 3:**
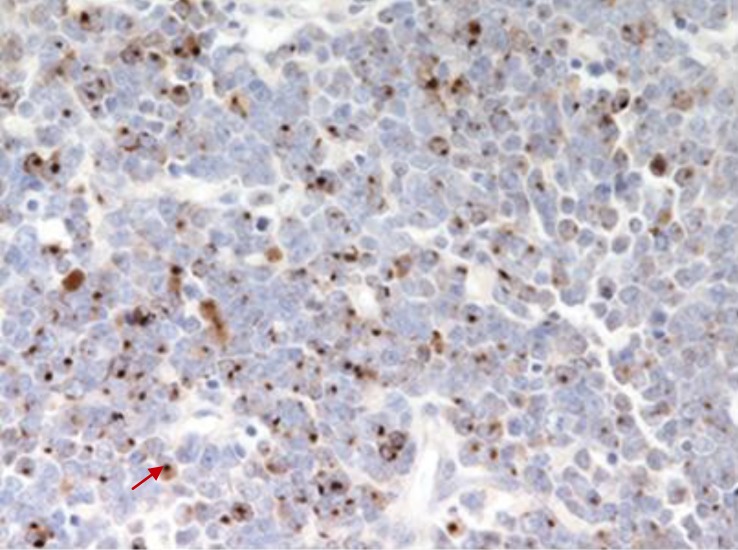
Prolactin immunoreactivity is evident in the cytoplasm, with peri-nuclear dot-like positivity of tumour cells (red arrow). Magnification 40×.

Post-operative histopathology, immunohistochemistry results under haematoxylin and eosin stain showed that the ovary was replaced by diffuse sheets of undifferentiated, highly atypical cells possessing enlarged nuclei. Tumour cells were positive for synaptophysin, p53 and epithelial membrane antigen (EMA), and showed negative expression for chromogranin, CK7, desmin, inhibin, melanA, PLAP and CD56. Following immunohistochemistry, the tumour was categorised as ovarian malignant small cell tumour of pulmonary type. Immunohistochemical staining for prolactin showed patchy but intense cytoplasmic, peri-nuclear dot-like positivity, which was reported to perhaps represent localisation of prolactin in Golgi complex (Fig. 3).

This patient had adjuvant chemotherapy following her surgery. Her PET FDG scan performed 10 weeks post-operatively showed right oophorectomy, unremarkable upper abdominal viscera, clear lung fields and no evidence of mediastinal, abdominal or pelvic adenopathy. She had no disease recurrence on her 8-month follow-up scan. Her prolactin levels remained normal.

## Discussion

Our case demonstrates ectopic prolactin secretion secondary to an ovarian malignant small cell tumour. Normalisation of prolactin levels soon after surgical removal of the tumour, positive immunohistochemistry for prolactin and normal pituitary gland on imaging strongly support the diagnosis. We report this case to highlight and make clinicians aware of this rare condition.

## Patient consent

Written informed consent was obtained from the patient/patient’s mother for publication of this case report.

## Author contribution statement

Co-authors have contributed towards patient care, management and also towards writing and finalising the draft.
